# Molecular imaging of EGFR and CD44v6 for prediction and response monitoring of HSP90 inhibition in an in vivo squamous cell carcinoma model

**DOI:** 10.1007/s00259-015-3260-x

**Published:** 2015-12-01

**Authors:** Diana Spiegelberg, Anja C. Mortensen, Ram K. Selvaraju, Olof Eriksson, Bo Stenerlöw, Marika Nestor

**Affiliations:** Department of Immunology, Genetics and Pathology, Uppsala University, Uppsala, Sweden; Unit of Otolaryngology and Head and Neck Surgery, Department of Surgical Sciences, Uppsala University, Uppsala, Sweden; Preclinical PET Platform, Uppsala University, Uppsala, Sweden

**Keywords:** HSP90 inhibitor, AT13387, ^124^I, PET, EGFR, CD44v6

## Abstract

**Purpose:**

Heat shock protein 90 (HSP90) is essential for the activation and stabilization of numerous oncogenic client proteins. AT13387 is a novel HSP90 inhibitor promoting degradation of oncogenic proteins upon binding, and may also act as a radiosensitizer. For optimal treatment there is, however, the need for identification of biomarkers for patient stratification and therapeutic response monitoring, and to find suitable targets for combination treatments. The aim of this study was to assess the response of surface antigens commonly expressed in squamous cell carcinoma to AT13387 treatment, and to find suitable biomarkers for molecular imaging and radioimmunotherapy in combination with HSP90 inhibition.

**Methods:**

Cancer cell proliferation and radioimmunoassays were used to evaluate the effect of AT13387 on target antigen expression in vitro. Inhibitor effects were then assessed in vivo in mice-xenografts. Animals were treated with AT13387 (5 × 50 mg/kg), and were imaged with PET using either ^18^F-FDG or ^124^I-labelled tracers for EGFR and CD44v6, and this was followed by ex-vivo biodistribution analysis and immunohistochemical staining.

**Results:**

AT13387 exposure resulted in high cytotoxicity and possible radiosensitization with IC_50_ values below 4 nM. Both in vitro and in vivo AT13387 effectively downregulated HSP90 client proteins. PET imaging with ^124^I-cetuximab showed a significant decrease of EGFR in AT13387-treated animals compared with untreated animals. In contrast, the squamous cell carcinoma-associated biomarker CD44v6, visualized with ^124^I-AbD19384 as well as ^18^F-FDG uptake, were not significantly altered by AT13387 treatment.

**Conclusion:**

We conclude that AT13387 downregulates HSP90 client proteins, and that molecular imaging of these proteins may be a suitable approach for assessing treatment response. Furthermore, radioimmunotherapy targeting CD44v6 in combination with AT13387 may potentiate the radioimmunotherapy outcome due to radiosensitizing effects of the drug, and could potentially lead to a lower dose to normal tissues.

**Electronic supplementary material:**

The online version of this article (doi:10.1007/s00259-015-3260-x) contains supplementary material, which is available to authorized users.

## Introduction

Head and neck squamous cell carcinoma (HNSCC) is the fifth most common solid cancer, with more than 500,000 new cases diagnosed worldwide every year [1]. Current treatment options for HNSCC are surgery, radiation with or without adjuvant chemotherapy and antibody therapy. With the exception of HPV-induced cancer, HNSCC is still a malignancy with a high risk of both residual and recurrent disease [2]. This demonstrates the need for earlier diagnosis and additional treatment options to target the disease more effectively.

Recent developments in fields such as antigen screening, protein engineering, and cancer biology have facilitated the rational design of targeted pharmaceuticals. However, for optimal treatment there exist a need for identification of biomarkers for patient stratification and therapeutic response monitoring, and to find suitable targets for combination treatments. Here, radioimmunotargeting that combines the high sensitivity and resolution of, for example, a PET camera with the tumour specificity of an antibody-based molecule, plays an important role. Based on essential information about potential treatment outcomes and from knowledge of the underlying molecular mechanisms, this may enable more personalized cancer treatment without invasive procedures [3].

One recently investigated target protein is the molecular chaperone heat shock protein 90 (HSP90), which is overexpressed in several haematological and solid tumours, including HNSCC [4]. Active HSP90 is essential for the activation of numerous client proteins that are involved in all hallmarks of cancer [5]. If HSP90 is inhibited, client proteins are ubiquitinated and finally destroyed by proteasomal degradation [6]. Thus, targeting and inhibition of HSP90 offers the unique possibility of overcoming mutations in downstream signalling proteins and of shutting down several pathways simultaneously [7]. In order to find a potential biomarker for early response to HSP90-targeted therapies, several studies have investigated the effects of first-generation HSP90 inhibitors (e.g. 17-AAG, 17-DMAG, NVP-AUY922) on HER2 expression [8–10]. However, HER2 expression in HNSCC is negligible and cannot be used as a biomarker for treatment outcomes in this patient group. Instead, overexpression of EGFR is common in HNSCC patients [4, 11], indicating that targeting EGFR could be a suitable approach. Wild-type EGFR has been shown to be a client protein of HSP90 [12] suggesting that their interaction is vital for the proliferation of EGFR-dependent cancers. Additionally, inhibition of the HSP90 chaperone function not only affects new EGFR protein synthesis: a recent study has demonstrated that fully mature, membrane-bound EGFR is stabilized by HSP90 independent of HER2 and that HSP90 inhibition leads to downregulation of mature EGFR [4].

The small-molecule inhibitor of HSP90 AT13387 (onalespib) is a novel second-generation synthetic non-ansamycin inhibitor currently in clinical trials. It inhibits the chaperone function of HSP90 and promotes degradation of oncogenic proteins upon binding [13]. AT13387 may also act as a radiosensitizer since HSP90 client proteins are involved in DNA repair mechanisms. Thus, this molecule is also attractive for possible combination treatments with other suitable therapeutic modalities such as radiotherapy, internal or external (including external beam radiation and radioimmunotherapy). However, given that HSP90 has more than 200 client proteins [14], it is of great importance to find suitable antigens for radioimmunotherapy that are not affected by HSP90 downregulation.

One potential target for radioimmunotherapy is the squamous cell carcinoma (SCC)-associated cell surface molecule CD44v6. This antigen has been found to be overexpressed in over 90 % of HNSCC, as well as in other cancers such as those of the lung, oesophagus and breast, which makes it an attractive target for molecular imaging and targeted therapy [15]. CD44v6 has been suggested to be involved in aggressive tumour behaviour as a tumour metastasis-promoting protein, and has been associated with a worse prognosis in several cancers including HNSCC [16]. Furthermore, in a recent study CD44v6 expression was not significantly altered by AT13387 treatment in vitro, making it a promising candidate for radioimmunotherapy in combination with HSP90 inhibition [17].

Consequently, the main goal of this study was to assess the response of the biomarkers EGFR and CD44v6 to AT13387 therapy by PET imaging in SCC. Studies were performed in vitro using cultured tumour cells and in vivo using xenografts in mice, with the aim of establishing not only reliable monitoring of HSP90 inhibition therapy in HNSCC, but also of combining such therapy with other suitable therapeutic modalities such as radioimmunotherapy.

## Materials and methods

### Cancer cell culture

The human SCC cell line A431 (EGFR++/CD44v6++) was obtained from the American Type Culture Collection (Manassas, VA) and the cells were cultured in Ham’s F10 medium supplemented with 10 % fetal calf serum, 2 mM l-glutamine, and antibiotics (100 IU penicillin and 100 μg/ml streptomycin). The HNSCC cell line UM-SCC-74B (EGFR+/CD44v6+) was kindly provided by Prof. T.E. Carey, University of Michigan, USA, and the cells were cultured in Dulbecco’s modified Eagle’s medium (DMEM), with the same supplements as above as well as 1 % non-essential amino acids. Cells of both cell lines were incubated at 37 °C in an atmosphere containing 5 % CO_2_. The cells used in monolayer culture experiments were detached using trypsin and seeded in separate dishes used for experiments 2 days prior to the studies. The cell lines in this study were cultured for less than 6 months after delivery.

### Drug and radiation treatment

AT13387 (Astex Pharmaceuticals, Cambridge, UK) was stored as a lyophilized powder and dissolved in 17.5 % (w/v) hydroxypropyl-β-cyclodextrin before use.

#### In vitro experiments

Cells seeded for cell viability (MTT assay, IC_50_) were preplated and incubated with AT13387 (0.01, 0.03, 0.1, 0.3, 1, 3, 10, 30, 100 μM). Radiation treatment was given 1 h after drug incubation using a ^137^Cs γ-ray irradiator at a dose of 1 Gy/min (Gammacell® 40 Exactor; Best Theratronics, Ottawa, ON, Canada). Cells seeded for radioimmunoassays were treated with 200 nM AT13387 for 24 h before analysis.

#### In vivo experiments

Mice in the treatment groups were given five daily injections of 50 mg/kg AT13387 (dissolved in 17.5 % w/v hydroxypropyl-β-cyclodextrin) subcutaneously into the neck area before small-animal imaging studies.

### MTT assay

The colorimetric MTT (3-(4,5-dimethylthiazol-2-yl)-2,5-diphenyltetrazolium bromide) assay (Vybrant® MTT cell proliferation assay kit; Life Technologies, Stockholm, Sweden) was used to determine viable cells after AT13387 and radiation treatment according to the manufacturer’s instructions. In brief, defined amounts of A431 and UM-SCC-74B cells were seeded into a 96-well plate in medium containing no phenol red and treated with AT13387 (at 0.01, 0.03, 0.1, 0.3, 1, 3, 10, 30 and 100 μM). After exposure to 10 μl of MTT solution for 4 h, cells were incubated with 100 μl of SDS-HCl solution for another 4 – 18 h. The plate was then read at 570 nm (yellow absorbance) using a plate reader (Bio-Rad, Sundbyberg, Sweden).

### Antibodies

The chimeric monoclonal antibody cetuximab, which recognizes the extracellular domain of EGFR and is FDA-approved for HNSCC treatment [4], was obtained from Merck (Darmstadt, Germany). Upon receipt, the antibody was purified from salts and amino acids by size exclusion chromatography on a PD-10 column (GE Healthcare, Uppsala, Sweden) and lyophilized. AbD19384 recognizes the cell surface antigen CD44v6, and is a recombinant bivalent antibody fragment engineered from two fully human monovalent AbD15179 Fab fragments. This bivalent format is functionally equivalent to a F(ab′)_2_ fragment. The generation of AbD15179 has been described previously [18]. AbD19384 was supplied from Bio-Rad AbD Serotec (Puchheim, Germany) in 3 × PBS.

### ^124^I and ^125^I labelling of cetuximab and AbD19384

Labelling of AbD19384 with ^124^I or ^125^I (both PerkinElmer, Waltham, MA) using 1,3,4,6-tetrachloro-3α,6α-diphenylglycouril (Iodogen) was performed as following; three Iodogen buffers (A, B, C) were prepared: A, 0.5 M sodium phosphate buffer; B, 0.05 M sodium phosphate and 5 M NaCl, pH 7.4; C, 0.05 M sodium phosphate, 5 % KI and 0.5 % BSA w/v, pH 7.4). Iodogen was dissolved in dichloromethane to 0.2 mg/ml. ^124^I or ^125^I was incubated with carrier iodide (cold NaI) at a molar ratio of 1:1 prior to starting the labelling procedure in order to improve labelling efficiency. Radioiodine along with AbD19384 or cetuximab (1 or 5 mg/ml in PBS, respectively) was added to tubes coated with 50 μg of Iodogen. Buffer A was added in an equivalent volume and incubated at room temperature for 7 min with shaking. The mixture was transferred to a new tube and buffer B (480 μl) was added. After 10 min incubation, buffer C (480 μl) was added and mixed thoroughly. Labelled conjugates were separated from nonreacted radionuclide and low molecular weight reaction components by using a NAP-5 or PD10 column (GE Healthcare, Uppsala, Sweden) pre-equilibrated with PBS. The yield, purity and stability of the labelled conjugates were determined by instant thin-layer chromatography (ITLC). Samples taken immediately and 48 h after the labelling procedure were analysed. Serum stability tests were performed by 1 h incubation at 37 °C in 42 % murine serum in PBS, pH 7.4. Approximately 1 μl of sample was then placed on an ITLC chromatography strip (Biodex) and placed in a “running buffer” (70 % acetone), followed by measurements on a Cyclone storage phosphor system (PerkinElmer, Waltham, MA). Data were analysed using OptiQuant image analysis software (PerkinElmer).

### Radioimmunoassays: in vitro binding, specificity and antigen density

A431 and UM-SCC-74B cells were seeded into 48-well plates and treated with 200 nM AT13387 for 24 h before washing with PBS and incubation with 0.1, 0.25, 1, 2.5, 5, 10, 15 or 30 nM ^124^I-cetuximab or ^124^I-AbD19384. Control wells were incubated with complete medium. After 4 h incubation at 37 °C cells were trypsinized, counted and measured in a gamma counter together with three standards of each concentration. The cell-bound activity in picomoles per 100,000 cells was calculated.

### Small-animal studies

#### Animal model

All experiments complied with current Swedish law and were performed with permission granted by the Uppsala Committee of Animal Research Ethics. Nu/nu Balb/c mice (19 mice, female) were housed under standard laboratory conditions and ad libitum access to feed. Approximately 8 × 10^6^ A431 cells (high EGFR and CD44v6 expression) suspended in 150 μl 1:1 cell medium/matrigel were injected subcutaneously into the left back leg, and approximately 5 × 10^6^ UM-SCC-74B cells (low EGFR and CD44v6 expression) into the right back leg of the same nude balb/c (nu/nu) mice. After inoculation, the weight of the animals and size of the tumours were monitored on alternate days. Mice bearing tumours of approximately 1 cm in diameter were used in the studies 2 weeks after injection.

#### Tracer injection, specific activity and ex vivo measurements

Animals were divided into an untreated/control group (*n* = 10) and a treatment group (*n* = 9). The groups were injected with either 16 μg ^124^I-labelled cetuximab (*n* = 6; injected activity 1.95 MBq/mouse, specific activity 122 kBq/μg cetuximab) or 11 μg ^124^I-labelled AbD19384 (*n* = 6; injected activity 1.5 MBq/mouse, specific activity of 136 kBq/μg) intravenously via a tail vein. Seven mice (four control, three treated) were injected with 4.0 ± 1.8 MBq ^18^F-FDG. In each group, one to three animals were used for small-animal PET/CT imaging at 48 h after injection (p.i.) for ^124^I-cetuximab and ^124^I-AbD19384 and at 30 min p.i. for ^18^F-FDG. All animals were killed with a mixture of ketamine and xylazine followed by heart puncture. Tumours, blood and tail (injection site) were collected, weighed and measured in a gamma counter, together with three injection standards of ^124^I-cetuximab and ^124^I-AbD19384. Radioactivity uptake in the organs was calculated as percent of injected dose per gram of tissue (%ID/g).

#### Small-animal PET/CT

One day before conjugate injections, mice were given potassium iodide (1 %) in their drinking water to block uptake of free ^124^I in the thyroid. Whole-body PET/CT studies were performed under general anaesthesia (isoflurane 1.0 – 2.5 % in 50 %/50 % medical oxygen/air at 450 ml/min) at 48 h p.i. for ^124^I-labelled cetuximab and AbD19384, and at 30 min p.i. for ^18^F-FDG. Mice with preinjected tracer were placed under sedation in the gantry of a small-animal PET/CT scanner (Triumph® trimodality system; TriFoil Imaging, Northridge, CA) and a whole-body PET scan was performed for 80 min in list mode followed by a CT scan for 3 min (field of view 8.0 cm). The ^18^F-FDG scan was performed for 60 min. The breathing rate was monitored with a camera under controlled anaesthesia (isoflurane 1.0 – 2.5 % in 50 %/50 % medical oxygen/air at 450 mL/min). Animals were placed on the heated bed of the small-animal PET scanner to prevent hypothermia and taped to prevent large movements during the study.

The PET data were reconstructed into a static image using an ordered subsets expectation maximization (OSEM) 3-D algorithm (20 iterations). The CT raw data were reconstructed using filtered back projection. PET data were reconstructed for attenuation and scatter correction with their respective CT data. PET and CT dicom files were analysed using PMOD v3.508 (PMOD Technologies Ltd, Zurich, Switzerland). Volumes of Interest were drawn manually on the tumours. Tracer uptake was quantified as the quotient between tumours with high and tumours with low EGFR/CD44v6 expression.

### Ex vivo immunohistochemistry

A431 and UM-SCC-74B tumours were fixed in formalin directly after dissection. Tumours were paraffin-embedded, sectioned and deparaffinized. Antigens were retrieved by microwaving (10 + 15 min) in citrate buffer (DAKO, S2369) or Tris-EDTA buffer (DAKO, S2367). Sections were immunostained with anti-HSP90 (abcam, UK), anti-CD44v6 (AbD Serotec) and anti-EGFR (Abcam, UK) according to he manufacturers’ instructions followed by detection using an EnVision FLEX system (DAKO, K8000). The reaction was visualized using EnVision FLEX DAB+ (DAKO). Mayer’s haematoxylin (DAKO) was used as counterstain. Images of the immunostained sections (magnification ×10) were obtained using a Nikon D3000 digital camera mounted on an inverted Nikon Diaphot-TMD microscope.

Immunohistochemistry assays were semiquantified according to the H-score method described previously [13, 19]. In brief, the H-score is acquired by manual scoring of each cell in five intensity groups: *0* no staining, *1* weak staining, *2* moderate staining, *3* dark staining, *4* maximum staining. The H-score is then the sum of (0*p0) + (1*p1) + (2*p2) + (3*p3) + (4*p4), where p0, p1, p2, p3 and p4 are the percentages of cells in the corresponding groups, yielding a range of 0 – 400 for the H-score. The H-score was determined for 16 separate sections of each tumour tissue sample, counting 100 cells per section for a total of 1,600 scored cells per sample. The immunohistochemistry assays were scored blinded with respect to target and treatment.

### Statistical analysis curve fitting

Microsoft Office Excel 2011 for Mac (Microsoft, Redmond, WA) and GraphPad Prism 6 for Mac (GraphPad Software, San Diego, CA) were used for data processing, graph plotting and statistical analysis. For the IC_50_ analysis and curve fitting, a normalized log (response) inhibition model was used, with a fixed Hill slope of −1: *Y* = 100/(1 + 10^((*X* − logIC_50_))), where *Y* is percent survival, *X* is drug concentration, and IC_50_ is drug concentration at 50 % survival. No parameter constraints were used. The error is represented as 95 % confidence interval. The significance of differences in cell viability between unirradiated and irradiated cells treated with AT13387 was assessed using a two-tailed paired *t*-test. For the in vivo specificity studies, the differences in ^124^I-cetuximab, ^124^I-AbD19384 and ^18^F-FDG uptake between tumours of control and AT13387 treatment groups were assessed using a two-tailed paired *t* test and were considered statistically significant for *P* < 0.05. The data are presented as means ± standard deviation (SD). The immunohistochemistry assays were scored using the H-score method (see above). A one-way ANOVA with Tukey’s post hoc test was used to evaluate the significance of differences in H-scores.

## Results

### Cell viability (IC_50_) after exposure to AT13387 and 2 Gy external radiation

**Fig. 1:**
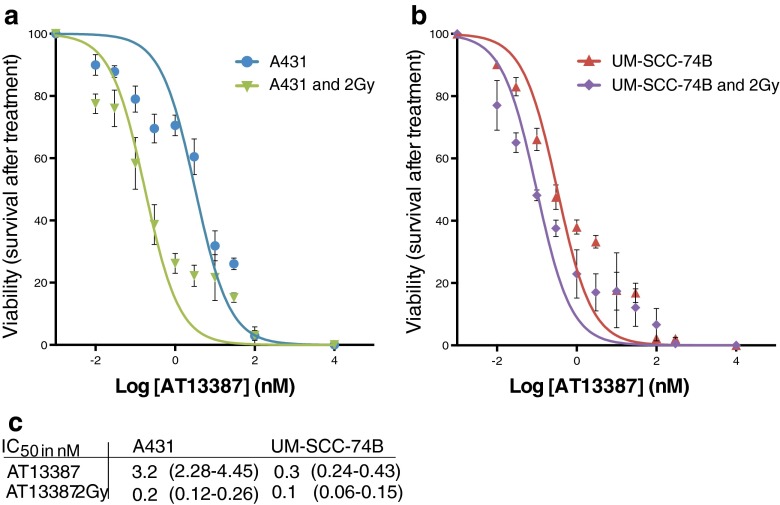
Cell survival in **a** A431 and **b** UM-SCC-74B cell lines after treatment with ten different concentrations of AT13387, with and without 2 Gy gamma radiation. Curves for A431 and UM-SCC-74B are normalized to untreated control cells and curves for A431 2 Gy and UM-SCC-74B 2 Gy to irradiated control cells. **c** IC_50_ values in nanomoles for AT13387 with 95 % confidence intervals in parentheses (*n* > 3)

To assess AT13387 treatment efficacy, MTT assays with various drug concentrations and/or external beam radiation were performed (Fig. 1). It is important to note that in Fig. 1a, b curves for A431 and UM-SCC-74B are normalized to survival at 0 nM AT13387 and curves “A431 and 2 Gy” and “UM-SCC-74B and 2 Gy” are normalized to survival at 0 nM AT13387 in cells irradiated with 2 Gy, in order to visualize the drug-related effects only. AT13387 treatment showed high cytotoxicity with IC_50_ values of 3.2 nM and 0.3 nM for A431 and UM-SCC-74B, respectively. Furthermore, all tested doses of AT13387 efficiently radiosensitized the cells of both cell lines. Combination treatment with the HSP90 inhibitor and a single radiation dose of 2 Gy decreased the IC_50_ value by a factor of three to 0.1 nM in UM-SCC-74B cells. The radiosensitizing effect of the drug was even greater for A431 cells in which a 16 times lower dose of AT13387 (IC_50_ 0.2 nM) reduced cell viability by 50 %. IC_50_ values for drug and radiation treatment together with 95 % confidence intervals are shown in Fig. 1c. The paired *t*-test demonstrated significant differences in cell viability between unirradiated and irradiated cells (*p* = 0.0053 and *p* = 0.0076 for A431 and UM-SCC74B cells, respectively).

### In vitro binding specificity and antigen density

**Fig. 2:**
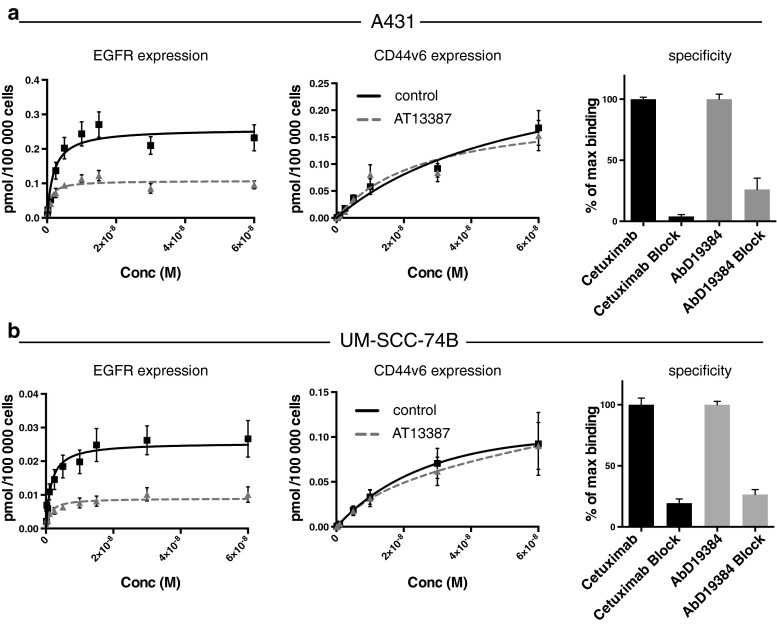
Expression of EGFR and CD44v6 and binding specificity in **a** A431 and **b** UM-SCC-74B cells using radioimmunoanalysis. Cells were exposed to 0.01 to 60 nM of ^124^I/^125^I-cetuximab or ^124^I/^125^I-AbD19384 and a 100-fold excess of unlabelled antibody was added at the highest concentrations to correct for nonspecific binding. The cells were counted and radioactivity measured in a gamma counter (*n* = 3, *error bars* SD)

Radioimmunoassays using the radiolabelled anti-EGFR antibody cetuximab and the radiolabelled anti-CD44v6 fragment AbD19384 were used to determine the expression of the cell surface markers EGFR and CD44v6, and demonstrated that A431 cells expressed both markers to a high extent (EGFR++/CD44v6++) and UM-SCC-74B cells expressed a lower amount (EGFR+/CD44v6+; for labelling and stability of the conjugates, see section Labelling and serum stability). Treatment with AT13387 significantly reduced EGFR expression by 58 % in A431 cells with high EGFR expression and by 64 % in UM-SCC-74B cells with low EGFR expression. CD44v6 expression was not significantly altered in either the cells with high CD44v6 expression or the cells with low CD44v6 expression (Fig. 2). Furthermore, binding of iodinated cetuximab and iodinated AbD19384 was blocked in both cell lines by an excess of unlabelled agent, demonstrating specific binding of the conjugates.

### Labelling and serum stability

Labelling yields for ^125^I-AbD19384 and ^125^I-cetuximab were 46 % and 74 %, respectively. Labelling yields for ^124^I-AbD19384 and ^124^I-cetuximab were 64 % and 72 %, respectively. Purity of all conjugates after size-exclusion chromatography was >95 %. The specific activities of the injection solutions for ^124^I-AbD19384 and cetuximab were 136 kBq/μg and 122 kBq/μg, that resulted in injected activity doses of 1.5 MBq (11 μg) and 1.95 MBq (16 μg) per mouse, respectively. Radiochemical purity of labelled conjugates stored in PBS for 48 h, or in serum for 1 h, was unchanged as shown by ITLC analysis.

### Small-animal PET/CT

**Fig. 3:**
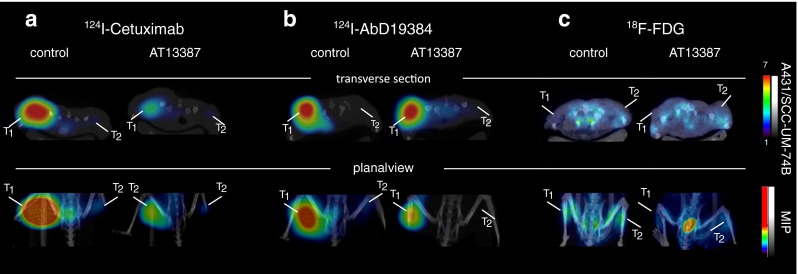
Representative small-animal PET/CT images of A431 tumours with high EGFR and CD44v6 expression (*T*_*1*_ left posterior flank) and UM-SCC-74B tumours with low EGFR and CD44v6 expression (*T*_*2*_ right posterior flank) in nude mice after intravenous injection of **a** ^124^I-cetuximab, **b** ^124^I-AbD19384, and **c** ^18^F-FDG. The *upper row* shows representative cross sections of the xenografts. The *lower row* shows planar maximum intensity projection images of the tracer distribution

Small-animal PET/CT imaging was used to trace the biodistribution of ^124^I-radiolabelled anti-EGFR cetuximab and ^124^I-radiolabelled anti-CD44v6 AbD19384 in comparison with the clinical standard ^18^F-FDG in a mouse xenograft model. Each mouse was carrying two tumours: an A431 tumour with high EGFR/CD44v6 expression in the left flank and a UM-SCC74B tumour with low EGFR/CD44v6 expression in the right flank. Animals in the treatment group received 50 mg/kg AT13387 on five consecutive days before PET/CT scanning. ^124^I-Cetuximab clearly distinguished between tumours with high and tumours with low EGFR expression, demonstrating the in vivo specificity of the conjugate. Furthermore, in the tumours with high EGFR expression, a clear reduction in tumour ratio was seen in the AT13387 treatment group (Fig. 3a). The anti-CD44v6 tracer ^124^I-AbD19384 successfully imaged the tumours with high expression in the control group tumours and the treatment group. For CD44v6, antigen expression was not significantly changed (Fig. 3b). Additionally, ^18^F-FDG PET analysis was not able to reveal the difference in receptor expression between treated and untreated mice xenografts imaged 30 min after ^18^F-FDG injection. The ^18^F-FDG uptake intensities were similar in the control and AT13387-treated mice (Fig. 3c). Furthermore, the ^18^F-FDG scans showed nonspecific uptake in brown fat in the neck/shoulder area (data not shown).

### Ex vivo measurements: tumour sizes and tracer uptake

**Fig. 4:**
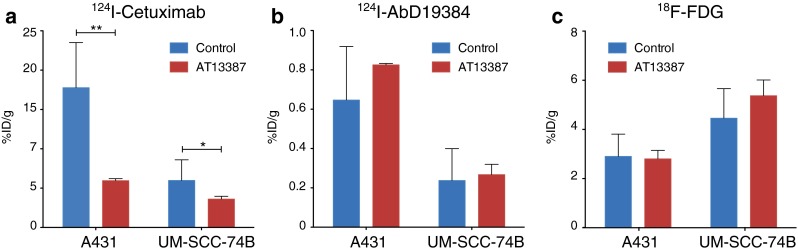
Tumour uptake of **a** ^124^I-Cetuximab (*n* = 6, 48 h after injection), **b** ^124^I-AbD19384 (*n* = 6, 48 h after injection) and **c** ^18^F-FDG (*n* = 7, 1 h after injection) in A431 tumours with high EGFR/CD44v6 expression and in UM-SCC-74B tumours with low EGFR/CD44v6 expression in control and AT13387-treated animals

Excised tumours were measured and weighed and tracer uptake was measured in a gamma counter. As expected, AT13387 treatment showed limited effects on the volume of A431 and UM-SCC-74B tumours. The changes in tumour size were not statistically significant, probably because of the short treatment time of 5 days (Supplementary Fig. 1). ^124^I-Cetuximab, ^124^I-AbD19384 and ^18^F-FDG uptake in the tumours with high and tumours with low EGFR/CD44v6 expression is shown in Fig. 4. ^124^I-Cetuximab uptake measurements demonstrated that AT13387 significantly reduced EGFR expression in tumours with high expression and tumours with low expression by 67 % (*p* < 0.001) and 40 % (*p* < 0.05), respectively (Fig. 4a). The expression of CD44v6, measured by ^124^I-AbD19384, was not significantly altered (Fig. 4b). Glucose metabolism in the tumours as measured by ^18^F-FDG uptake was unchanged in control and treatment groups (Fig. 4c).

### Ex vivo immunohistochemistry

**Fig. 5:**
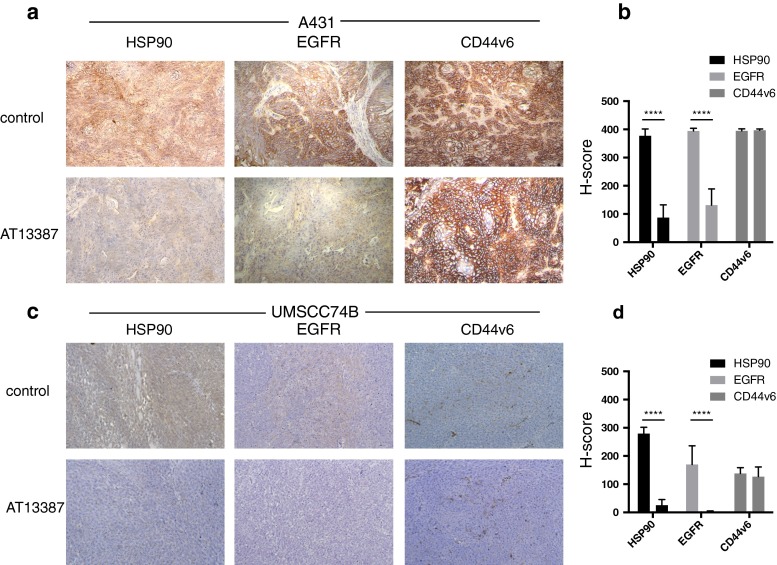
**a** **c** Ex vivo immunohistochemical staining for HSP90, EGFR and CD44v6 expression on representative sections of **a** A431 and **c** UM-SCC-74B tumour xenografts (×10). A431 tumours show high expression and UM-SCC-74B tumours show low expression of EGFR and CD44v6. HSP90 and EGFR were downregulated in the AT13387 treatment group. CD44v6 expression was unchanged. **b**, **d** Semiquantitative analysis of immunostaining using the H-score (*n* = 16, *error bars* SD; ****p < 0.0001, one-way ANOVA with Tukey’s post hoc test)

Ex vivo immunohistochemistry staining showed no major morphological differences between tumours from the control and AT13387-treated animals (Fig. 5a, c). A431 tumours (EGFR++/CD44v6++) and UM-SCC-74B tumours (EGFR+/CD44v6+) contained viable tumour and stromal cells, well-established blood vessels and minor areas of necrosis. Untreated A431 cells showed overexpression of the biomarkers EGFR and CD44v6, but untreated UM-SCC-74B cells showed only low expression. AT13387 treatment resulted in a strong reduction in the target protein HSP90 and client protein EGFR in both tumour models as shown by the H-scoring method, while no significant changes in CD44v6 expression were observed (Fig. 5b, d).

## Discussion

HSP90 client proteins are involved in all hallmarks of cancer, making HSP90 inhibitors promising as anticancer drugs. This study focused on the novel HSP90 inhibitor AT13387, and potential biomarkers for monitoring outcomes of AT13387 treatment with and without radioimmunotargeting. A thorough understanding of the molecular properties in different tumour types, before, during and after treatment, is essential to form a basis for personalized – and consequently more effective – patient management. Studies such as that presented here are thus absolutely necessary to realize this goal, and this study can be seen as a first step towards personalized cancer therapy.

AT13387 is a promising therapeutic drug currently in clinical trials for several cancer types including HNSCC [6, 20]. Toxicity data show that 120 mg/m^2^/dose is the maximum tolerated dose in patients with advanced solid tumours based on the incidence of moderate toxicity [6]. However, only moderate effects have been obtained so far in clinical trials with AT13387 as the sole agent in patients with advanced solid tumours [6, 20]. In the present study, we demonstrated in vitro that AT13387 is indeed very potent with an IC_50_ value more than 20 times lower than that of the well-characterized HSP90 inhibitor 17-AAG. As expected with the short treatment time assessed in vivo, AT13387 treatment showed limited effects on tumour volume, with no statistically significant changes in tumour size (Supplementary Fig. 1). The choice of end-point in the present study was selected in order to measure rapid, transient effects on protein expression over the initial days after treatment, not primarily to obtain therapeutic effects. It is therefore possible that in vivo a treatment effect could be obtained with other doses and treatment times.

For AT13387, clinical trials are currently in progress assessing the efficacy of combination treatments, e.g. AT13387 in combination with crizotinib in the treatment of non-small-cell lung cancer, in combination with imatinib in patients with gastrointestinal stromal tumour, and in combination with paclitaxel in patients with breast cancer. Furthermore, a clinical study assessing AT13387 in HNSCC patients receiving radiation therapy and cisplatin is one of several interesting clinical trials in the pipeline [6, 21]. In the present study, we assessed the potential benefit of combining AT13387 with radiotherapy, with promising results. The efficacy of the combination treatment was significantly greater than with AT13387 alone and radiation treatment alone, suggesting that the drug has radiosensitizing effects (Fig. 1). These effects have the potential to be utilized in conventional external beam radiotherapy or in a radioimmunotherapy, and raise expectations for the impending clinical trials combining AT13387 with radiotherapy, and for future combinations with radioimmunotherapy.

The possibility to noninvasively assess treatment efficacy during ongoing therapy is likely to be an important factor in estimating the duration of the treatment effect on cellular events and for defining a personalized drug dose and schedule [10]. Repeated PET scanning is especially important in targeted cancer treatments, since the drug-induced effects on biomarkers are often temporary and not easily assessed by invasive methods such as tumour biopsy sampling. For treatment response monitoring, we studied the effect of AT13387 on the client protein EGFR. In vitro radioimmunoassays with cetuximab demonstrated that AT13387 exposure reduced the receptor expression in SCC cells with high and cells with low EGFR expression (Fig. 2). This was in line with our results in vivo demonstrating that molecular imaging using PET can be used to noninvasively monitor changes in EGFR expression as a response to treatment with AT13387 (Fig. 3). These results were further confirmed by biodistribution analysis that showed that AT13387 significantly reduced EGFR expression in tumours with high expression and tumours with low expression (Fig. 4), and in ex vivo immunohistochemistry stainings (Fig. 5).

As expected, ^18^F-FDG PET was not able to reveal the difference in receptor expression of HSP90 client proteins after HSP90 inhibition (Figs. 3 and 4). This is in line with the findings of previous studies, in which, for example, administration of the HSP90 inhibitor NVP-AUY922 to multicellular breast cancer spheroids did not cause significant changes in ^18^F-FDG uptake [22]. Also, ^18^F-FDG PET can give false-positive results in areas of inflammation and brown fat, demonstrating the importance of specific targets and tracers for molecular imaging to assess the effects of HSP90 inhibitors. Thus, our results suggest that the use of EGFR-specific PET for AT13387 treatment response assessment could be a promising approach.

Besides external radiotherapy, combination treatment with AT13387 and radioimmunotherapy could benefit patients due to the radiosensitizing effects of the drug, and may overcome resistance mechanisms. Given that HSP90 has more than 200 client proteins [14], it is of great importance to find biomarkers that are not affected by HSP90 downregulation. To investigate possible new targets for radioimmunotherapy in combination with HSP90 inhibition we studied the cell surface protein CD44v6, an oncogenic isoform of the hyaluronan binding molecule CD44 frequently overexpressed in HNSCC [15, 23]. In vivo, small-animal PET/CT scans of xenografts in mice using ^124^I-AbD19384 gave high-quality high-contrast images of high CD44v6-expressing tumours. No significant difference in ^124^I-AbD19384 uptake between control and treatment groups was detected with small-animal PET/CT (Fig. 3). In vitro studies with ^124^I-labelled AbD19384 confirmed that CD44v6 was not significantly affected by AT13387 (Fig. 2). These results were further confirmed by biodistribution (Fig. 4) and ex vivo immunohistochemistry (Fig. 5) where CD44v6 expression was similar in treated and untreated animals. Thus, our results demonstrate that CD44v6 could be a suitable target for radioimmunotherapy in combination with HSP90 inhibition.

In conclusion, the novel HSP90 inhibitor AT13387 showed radiosensitizing and cytotoxic activity and caused a significant decrease in EGFR expression both in vivo and in vitro while CD44v6 expression was unchanged. Our results demonstrate that EGFR imaging using PET could be a useful tool for monitoring treatment response and that CD44v6 could be a suitable target for radioimmunotherapy in combination with HSP90 inhibition.

## Electronic supplementary material

Below is the link to the electronic supplementary material.

Supplementary Fig. 1 Tumour weights in grams after dissection. There was no statistically significant difference between control and AT13387 treated A431 and UM-SCC-74B tumours (PDF 21 kb)
