# Supplementary figures and images for: Molecular imaging of EGFR and CD44v6 for prediction and response monitoring of HSP90 inhibition in an in vivo squamous cell carcinoma model
Source: Eur J Nucl Med Mol Imaging. 2015 Dec 1;43:974–82. doi: 10.1007/s00259-015-3260-x (PMC4819754; doi:10.1007/s00259-015-3260-x)

# Tumor size

weight in g

1.5  
1.0  
0.5  
0.0

Control A431

AT13 A431

Control 74B

AT13 74B

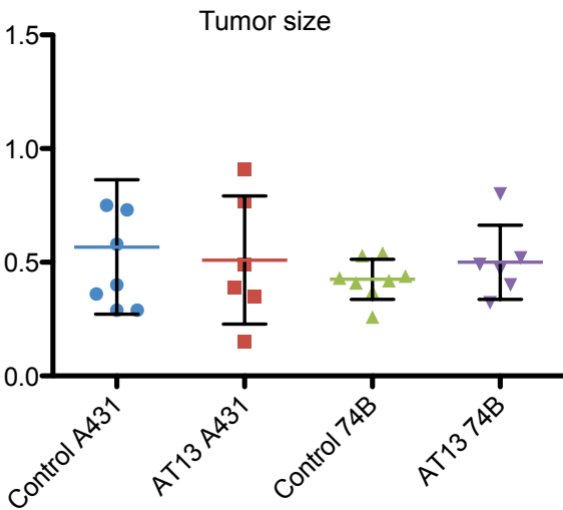

Supplement: Supplementary file 1 — Tumour weights in grams after dissection. There was no statistically significant difference between control and AT13387 treated A431 and UM-SCC-74B tumours (PDF 21 kb) [file 259_2015_3260_MOESM1_ESM.pdf]
